# Tau kinetics in Alzheimer's disease

**DOI:** 10.3389/fnagi.2022.1055170

**Published:** 2022-11-09

**Authors:** Daniel B. Hier, Sima Azizi, Matthew S. Thimgan, Donald C. Wunsch

**Affiliations:** ^1^Applied Computational Intelligence Laboratory, Department of Electrical & Computer Engineering, Missouri University of Science & Technology, Rolla, MO, United States; ^2^Department of Neurology and Rehabilitation, University of Illinois at Chicago, Chicago, IL, United States; ^3^Department of Biological Sciences, Missouri University of Science & Technology, Rolla, MO, United States; ^4^ECCS Division, National Science Foundation, Alexandria, VA, United States

**Keywords:** tau, Alzheimer's disease, CSF levels, plasma levels, steady state kinetics, halflife, turnover, clearance

## Abstract

The cytoskeletal protein tau is implicated in the pathogenesis of Alzheimer's disease which is characterized by intra-neuronal neurofibrillary tangles containing abnormally phosphorylated insoluble tau. Levels of soluble tau are elevated in the brain, the CSF, and the plasma of patients with Alzheimer's disease. To better understand the causes of these elevated levels of tau, we propose a three-compartment kinetic model (brain, CSF, and plasma). The model assumes that the synthesis of tau follows zero-order kinetics (uncorrelated with compartmental tau levels) and that the release, absorption, and clearance of tau is governed by first-order kinetics (linearly related to compartmental tau levels). Tau that is synthesized in the brain compartment can be released into the interstitial fluid, catabolized, or retained in neurofibrillary tangles. Tau released into the interstitial fluid can mix with the CSF and eventually drain to the plasma compartment. However, losses of tau in the drainage pathways may be significant. The kinetic model estimates half-life of tau in each compartment (552 h in the brain, 9.9 h in the CSF, and 10 h in the plasma). The kinetic model predicts that an increase in the neuronal tau synthesis rate or a decrease in tau catabolism rate best accounts for observed increases in tau levels in the brain, CSF, and plasma found in Alzheimer's disease. Furthermore, the model predicts that increases in brain half-life of tau in Alzheimer's disease should be attributed to decreased tau catabolism and not to increased tau synthesis. Most clearance of tau in the neuron occurs through catabolism rather than release to the CSF compartment. Additional experimental data would make ascertainment of the model parameters more precise.

## Introduction

The pathological hallmarks of Alzheimer's disease are extracellular amyloid plaques and intraneuronal neurofibrillary tangles. Abnormally phosphorylated tau is a component of neurofibrillary tangles. Tau is a highly soluble cytosolic protein that binds to microtubules and is largely confined to the neuron (Mandelkow and Mandelkow, 2012). In Alzheimer's disease, tau may undergo hyper-phosphorylation, truncation, and aggregation into oligomers. This can lead to insoluble fibrils (Orr et al., 2017). Tau is measurable in the brain, cerebrospinal fluid (CSF), brain interstitial fluid (ISF), and plasma by sensitive assay methods, including the single molecule array (Rissin et al., 2010; Han et al., 2017; Hier et al., 2021). Tau levels are elevated in the plasma, CSF, and brain of patients with Alzheimer's disease (Mattsson et al., 2016; Olsson et al., 2016; Koss et al., 2018; Fossati et al., 2019). Tau is measurable in the plasma and CSF of healthy subjects (**Table 2**) which suggests that tau is released from neurons into the ISF in health and in disease. Tau that is released by neurons into the ISF can exchange with the CSF and drain to the plasma (Hier et al., 2021). CSF levels of tau are generally 100 times higher than plasma levels. Correlations between plasma levels and CSF levels of tau may be weak (Fossati et al., 2019). Soluble cytosolic tau (as opposed to insoluble tau in neurofibrillary tangles) correlates with disease stage and cognitive deficits (Koss et al., 2018). While levels of tau are elevated in the plasma, CSF, and brain of patients with Alzheimer's disease, no consensus exists that tau is causative of Alzheimer's disease (Mandelkow and Mandelkow, 1998; Gendreau and Hall, 2013; Tarasoff-Conway et al., 2015; Josephs, 2017; Orr et al., 2017; Xin et al., 2018; Naseri et al., 2019; Harrison et al., 2020; Ishida et al., 2022). Furthermore, consensus is lacking on the cause of elevated tau levels in Alzheimer's disease. Rising levels of tau could be due to increased neuronal synthesis, increased neuronal release, decreased neuronal clearance, or impaired CSF clearance of tau (de Vrij et al., 2004; Yamada et al., 2011, 2014, 2015; Chai et al., 2012; Gendreau and Hall, 2013; Tarasoff-Conway et al., 2015; Orr et al., 2017; Merezhko et al., 2018; Vaz-Silva et al., 2018; Xin et al., 2018; Kitaguchi et al., 2019; Patel et al., 2019; Pernègre et al., 2019; Ruan and Ikezu, 2019; Strang et al., 2019; Brunello et al., 2020; Harrison et al., 2020; Nimmo et al., 2020; Liu et al., 2021; Ishida et al., 2022).

Kinetic equations can model levels of tau in brain, CSF, and plasma compartments. These equations can characterize the movement of tau from compartment to compartment and the rates at which tau is synthesized, cleared, released, and absorbed. Tau is successively cleared from three compartments: the brain (neuron), the CSF, and the plasma. Although prior work has examined the kinetics of neurological protein biomarkers (Reiber, 2001; Brophy et al., 2011; Dadas et al., 2016; Ercole et al., 2016; Moody et al., 2017; Thelin et al., 2017; Welch et al., 2017; Dadas and Janigro, 2018; Sato et al., 2018; Lehmann et al., 2019; Azizi et al., 2021), detailed kinetic models that relate the CSF and plasma levels of tau are not available. Isotopic methods have examined tau turnover in humans and animals (Yamada et al., 2014, 2015; Sato et al., 2018). We have previously used pharmacokinetic equations to model the rise and fall of protein biomarkers in the plasma after mild traumatic brain injury (Azizi et al., 2021). We propose that kinetic equations (Rosenbaum, 2016) can model tau levels in the brain, CSF, and plasma compartments after changes in the rates at which tau is synthesized, released, and cleared.

## Methods

### The model has three connected compartments

**Figure 1:**
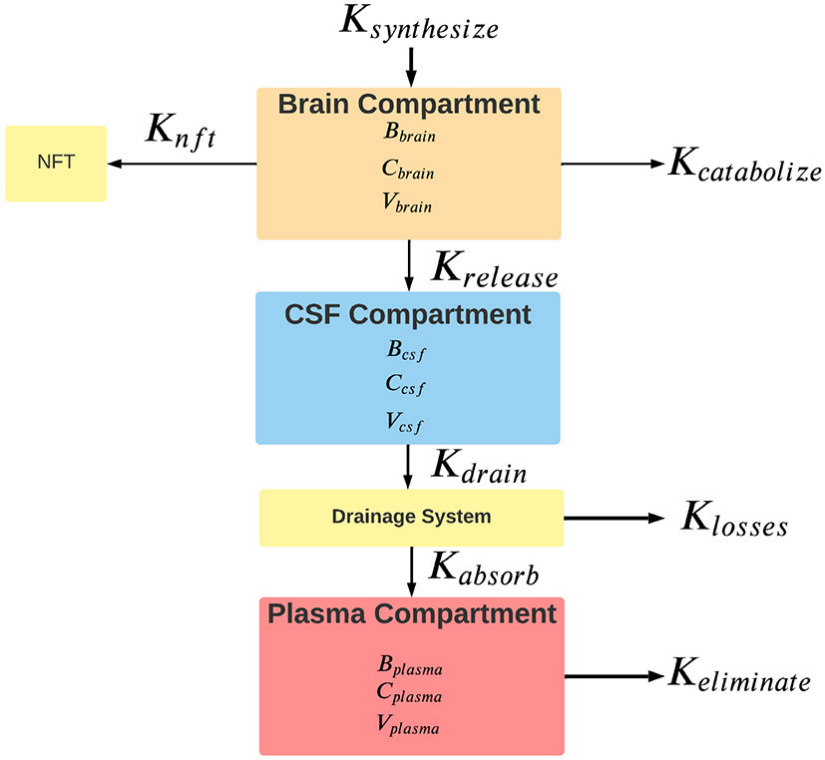
Rates of the flow of tau between model compartments. For each compartment, *B* is the total tau in pg, *C* is the tau concentration in pg/ml, and *V* is the compartment volume in ml. Rates are shown with a capital *K* and are nominally expressed in units of pg/hr. *K*_*synthesize*_ is the synthesis rate of tau, *K*_*catabolize*_ is the internal rate of tau catabolism (destruction) within neurons. *K*_*release*_ is the rate of tau release from neurons into the interstitial fluid. Some cytosolic tau is converted to neurofibrillary tangles (NFT) in the neuron at an unknown rate of *K*_*nft*_. Clearance of tau from the brain compartment is the sum of rates of catabolism, release, and conversion to neurofibrillary tangles. *K*_*drain*_ is the rate that tau enters the drainage system. The tau drainage system from the CSF likely encompasses a variety of mechanisms, including the arachnoid granulations of the CSF, the glymphatic system, connections between the CSF and olfactory and meningeal lymphatics, and the periarterial drainage system. Clearance of tau from the CSF reflects the contributions of all of these drainage systems to removing tau from the CSF. *K*_*losses*_ is the rate of loss of tau in the drainage system. *K*_*absorb*_ is the tau absorption rate into the plasma compartment. *K*_*eliminate*_ is the rate that tau is eliminated from the plasma compartment. Elimination from the plasma occurs through various mechanisms, including renal excretion, hepatic metabolism, and intravascular proteolysis. Clearance from the plasma reflects all of these elimination mechanisms.

The proposed model has three connected compartments: brain, CSF, and plasma (Figure 1). The total tau in each compartment is denoted by upper case B (*B*_*brain*_, *B*_*csf*_, and *B*_*plasma*_). Compartmental tau levels are denoted by upper case C (*C*_*brain*_, *C*_*csf*_, and *C*_*plasma*_). Compartmental volumes are denoted by upper case V (*V*_*brain*_, *V*_*csf*_, and *V*_*plasma*_). Rates are shown with an upper case K. Available tau in each compartment depends on the tau level and the volume of distribution in the compartment.


$$\begin{array}{l} \text{tau content in compartment}=\text{tau level}*\text{volume of distribution }\\ \text{ B}=\text{C}*\text{V}. \end{array}$$

### Model assumptions

The volumes of distribution for tau in the CSF and ISF are not known. As a model simplification we combined the ISF volume of 150 ml and the CSF volume of 150 ml into a single combined CSF compartment with an estimated volume of 300 ml (Lei et al., 2017; Fleischman and Berdahl, 2019; Shetty and Zanirati, 2020). It is assumed that tau release from neurons reflects normal biological processes, neurological disease, or response to injury (Pooler et al., 2013; Sorci et al., 2013; Yamada et al., 2014). After release by neurons, tau enters the ISF. Some released tau could enter drainage pathways without mixing with the CSF. Other released tau enters the cerebrospinal fluid (CSF) through multiple mechanisms (Hladky and Barrand, 2014; Bakker et al., 2016; Lei et al., 2017). Mixing of the ISF and CSF is incomplete so tau levels are higher in the ISF than in the CSF (Herukka et al., 2015). Tau plasma levels in the plasma are assumed to be homogeneous without gradients. Regional differences in tau levels in the brain are known (Han et al., 2017). However, as a model simplification tau levels are stipulated to be homogeneous within the brain. As a model simplification, we approximated the volume of the brain compartment as wet brain weight. Although brain weight varies by age and sex, we used an average value of 1,300 g (Molina and DiMaio, 2012, 2015). Given that 10% of wet brain weight is protein (Banay-Schwartz et al., 1992), we estimated total brain protein as 1.3*10^14^ pg or 130 g. As the brain is mostly solid, brain size is expressed in g. The volume of distribution of tau in the plasma is unknown. If tau was confined intravascularly until elimination, the volume of distribution (*V*_*plasma*_) would equal the plasma volume of 3,500 ml (Tarazi et al., 1969; Tobias and Ballard, 2022). We did not correct for known differences in plasma volume related to sex, height, and weight. Furthermore, as a model simplification, we did not consider the possibility of tau redistributing into the body interstitial fluid (Lönsmann Poulsen, 1974; Rutili and Arfors, 1977). Model variables *C*_*csf*_ and *C*_*plasma*_ are the tau levels in the CSF and plasma, respectively. Most studies have measured tau in the blood based on plasma. Evidence suggests that plasma and serum levels of protein biomarkers are equivalent although differences have been reported (O'Connell et al., 2019; Huebschmann et al., 2020). Tau levels can be measured in the ISF (usually by microdialysis), the ventricular CSF (usually by ventricular drain), or the lumbar CSF (usually after lumbar puncture). The model assumes that *C*_*csf*_ is based on lumbar CSF which is the most common site for sampling. Tau levels in the ISF are higher than the CSF (Herukka et al., 2015). As a simplification, we have assumed that lumbar CSF tau levels are representative of the entire CSF compartment.

It is assumed that plasma and CSF levels of tau are at a steady state in the short run. Several studies have shown that this assumption is valid and that diurnal variations in levels are not significant (Blennow et al., 2007; Slats et al., 2012; Le Bastard et al., 2013; Cicognola et al., 2016). In order to maintain steady-state levels of tau in the brain compartment, the rate of synthesis of tau (*K*_*synthesize*_) is matched to the rate of destruction (*K*_*catabolize*_) plus the rate of release into the CSF compartment (*K*_*release*_) plus the rate that tau is converted into an insoluble form in neurofibrillary tangles (1). An imbalance in these rates would result in rising tau levels. The release of tau from the brain compartment (*K*_*release*_) into the CSF compartment equals the rate of tau draining from the CSF (*K*_*drain*_), otherwise tau levels would change in the CSF compartment (2). At a steady state, the amount of tau entering the plasma compartment (*K*_*absorb*_ must equal the outflow of tau from the CSF compartment (*K*_*drain*_) minus losses of tau in the drainage pathways (*K*_*losses*_) (3). Finally, at a steady state, the rate of tau absorption into the plasma compartment (*K*_*absorb*_) equals the rate of tau elimination from the plasma compartment (*K*_*eliminate*_) (4).


(1) $$\begin{array}{l} K_{synthesize}=K_{catabolize}+K_{release}+K_{nft}. \end{array}$$

(2) $$\begin{array}{l} K_{release}=K_{drain}. \end{array}$$

(3) $$\begin{array}{l} K_{absorb}=K_{drain}-K_{losses}. \end{array}$$

(4) $$\begin{array}{l} K_{absorb}=K_{eliminate}. \end{array}$$

Not all tau that leaves the CSF compartment and enters the drainage pathways (Figure 1) reaches the plasma compartment. Some may be diverted to other compartments, some may undergo proteolysis en route, and some may be lost in transit. Nimmo et al. (2020) have shown that in animal models some tau injected into the brain substance is retained in perivascular macrophages which interferes with its entrance into the drainage pathways. The proportion of tau that reaches the plasma from the CSF compartment is denoted by F (the fractional absorption ratio) which is dimensionless and varies from 0 to 1 (Price and Patel, 2020). A value of 1 for F indicates that 100% of the tau that drained from the CSF compartment reached the plasma compartment and a value of 0 indicates that 0% of the drained tau reaches the plasma compartment. In analogy to drug pharmacokinetics (Rosenbaum, 2016), F reflects the bioavailability of tau in the CSF compartment to the plasma compartment. The higher the value of F, the greater the proportion of tau in the CSF compartment that is bioavailable to the plasma compartment. Since the source of tau absorbed into the plasma compartment is tau drained from the CSF compartment, the rate of absorption into the plasma compartment is reduced by the fractional absorption ratio (5).


(5) $$\begin{array}{l} K_{absorb}=F*K_{drain}. \end{array}$$

The model assumes that absorption rates and elimination rates follow first-order kinetics (Rosenbaum, 2016). This means that the rate at which tau is absorbed into a compartment or the rate at which tau is eliminated from a compartment is linearly related to the amount of tau available in the compartment times a rate constant (6).


(6) $$\begin{array}{l} K=k*B. \end{array}$$

Where K is the rate (nominally in pg/hr), k is the first-order rate constant (nominally in *hr*^−1^). and B is the compartmental tau content (nominally in pg). Our previous study of the rise and fall of blood biomarkers after mild traumatic brain injury suggests that the assumption of first-order kinetics is reasonable for absorption into the plasma compartment and elimination from the plasma compartment for several neurological biomarkers (Azizi et al., 2021). We have also assumed that synthesis of tau is a zero-order process (independent of tau levels in the neuron) and that tau release and tau catabolism are first-order processes (linearly related to tau levels in the neuron) (Rosenbaum, 2016; Ross et al., 2021).


(7) $$\begin{array}{l} K_{catabolize}=k_{c}*B_{brain}. \end{array}$$

(8) $$\begin{array}{l} K_{release}=k_{r}*B_{brain}. \end{array}$$

The rate of tau drainage from the CSF compartment and the rate of tau absorption into the plasma compartment can be expressed with first-order rate constants.


(9) $$\begin{array}{l} K_{drain}=k_{d}*V_{csf}*C_{csf}. \end{array}$$

(10) $$\begin{array}{l} K_{absorb}=k_{a}*V_{csf}*C_{csf}*F. \end{array}$$

*K*_*absorb*_ is the rate of absorption into the plasma compartment, *C*_*csf*_ is the concentration of tau in the CSF compartment, *V*_*csf*_ is the volume of distribution of tau in the CSF compartment, *k*_*a*_ is the first-order plasma absorption rate constant, and F is the fractional absorption rate ratio. The rate of elimination of tau from the plasma compartment can also be expressed with a first-order elimination constant.


(11) $$\begin{array}{l} K_{eliminate}=k_{e}*V_{plasma}*C_{plasma}. \end{array}$$

Since at a steady state in the plasma compartment, *K*_*absorb*_ = *K*_*eliminate*_, Equation (10) can be set equal to (11) which allows for a calculation of F.


(12) $$\begin{array}{l} F=\frac{k_{e}*V_{plasma}*C_{plasma}}{k_{a}*V_{csf}*C_{csf}}. \end{array}$$

Tau leaving the CSF compartment is the source of tau entering the plasma compartment. After substituting (9) and (10) into (5), the model predicts that the first-order elimination rate constant for tau from the CSF compartment is equal to the first-order absorption rate constant for the plasma compartment.


(13) $$\begin{array}{l} k_{d}=k_{a}. \end{array}$$

At steady state, *K*_*release*_ = *K*_*drain*_ which allows the calculation of *k*_*r*_.


(14) $$\begin{array}{l} k_{r}=\frac{k_{d}*V_{csf}*C_{csf}}{B_{brain}}. \end{array}$$

### Model parameters

The first-order plasma elimination constant *k*_*e*_ was calculated based on literature estimates of tau plasma half-life (Azizi et al., 2021) and the standard kinetic equation relating half-life to the first-order elimination constant (Rosenbaum, 2016).


(15) $$\begin{array}{l} k_{e}=\frac{0.693}{t_{\frac{1}{2}}}. \end{array}$$

The value of *k*_*e*_ was combined with literature derived values for *T*_*max*_ (Azizi et al., 2021) to calculate the plasma first-order absorption constant (Rosenbaum, 2016). Equation (16) was solved in Excel for *k*_*a*_ by approximate methods.


(16) $$\begin{array}{l} T_{max}=\frac{\ln\;\left(\frac{k_{a}}{k_{e}}\right)}{k_{a}-k_{e}}. \end{array}$$

Values for plasma half-life of tau and plasma *T*_*max*_ of tau after traumatic brain injury were from the published literature as previously described (Azizi et al., 2021). Tau cellular half-life in the brain was determined by isotopic methods in humans (Sato et al., 2018). Similar tau cellular half-life estimates are available from mouse studies (Fornasiero et al., 2018).

The values of F for healthy controls, minimal cognitive impairment subjects (MCI), and Alzheimer's disease subjects (AD) were calculated from (12). Values of *C*_*csf*_ and *C*_*plasma*_ are from Table 2. Values of *k*_*e*_ and *k*_*a*_ are from Table 1.

**Table 1:** Model variables and parameters.

**Entity**	**Description**	Value^*^	**Units**
*K*_*synthesize*_	Rate that tau is synthesized in neuron	9.70 * 10^7^	pg/hr/brain
*K*_*catabolize*_	Rate that tau is catabolized in neuron	9.70 * 10^7^	pg/hr/brain
*K*_*release*_	Rate that tau is released from neuron	7.08 * 10^3^	pg/hr/brain
*K*_*nft*_	Rate that soluble tau is converted to NFT	unknown	pg/hr/brain
*K*_*losses*_	Rate that tau is lost in drainage pathways	6.43 * 10^3^	pg/hr/brain
*K*_*drain*_	Rate that tau is drained from CSF	7.08 * 10^3^	pg/hr/brain
*K*_*absorb*_	Rate tau is absorbed into plasma	6.51 * 10^2^	pg/hr/brain
*K*_*eliminate*_	Rate tau is eliminated from plasma	6.51 * 10^2^	pg/hr/brain
*k*_*d*_	CSF first-order elimination rate constant	7.0 * 10^−2^	*hr* ^−1^
*k*_*r*_	Brain first-order release rate constant	9.2 * 10^−8^	*hr* ^−1^
*k*_*a*_	Plasma first-order absorption rate constant	7.0 * 10^−2^	*hr* ^−1^
*k*_*e*_	Plasma first-order elimination rate constant	6.9 * 10^−2^	*hr* ^−1^
*k*_*c*_	Brain first-order catabolism rate constant	1.26 * 10^−3^	*hr* ^−1^
*V*_*csf*_	Volume of CSF compartment	300	ml
*V*_*brain*_ ^‡^	Volume of brain compartment	1,300	g
*V*_*plasma*_	Volume of plasma compartment	3,500	ml
CSF half-life	Tau half-life in CSF compartment	9.9	h
Brain half-life^†^	Tau half-life in brain compartment	552	h
Plasma half-life	Tau half-life in plasma compartment	10	h
Plasma *T*_*max*_	Time to maximum tau plasma level after TBI	8	h

**Figure 2:**
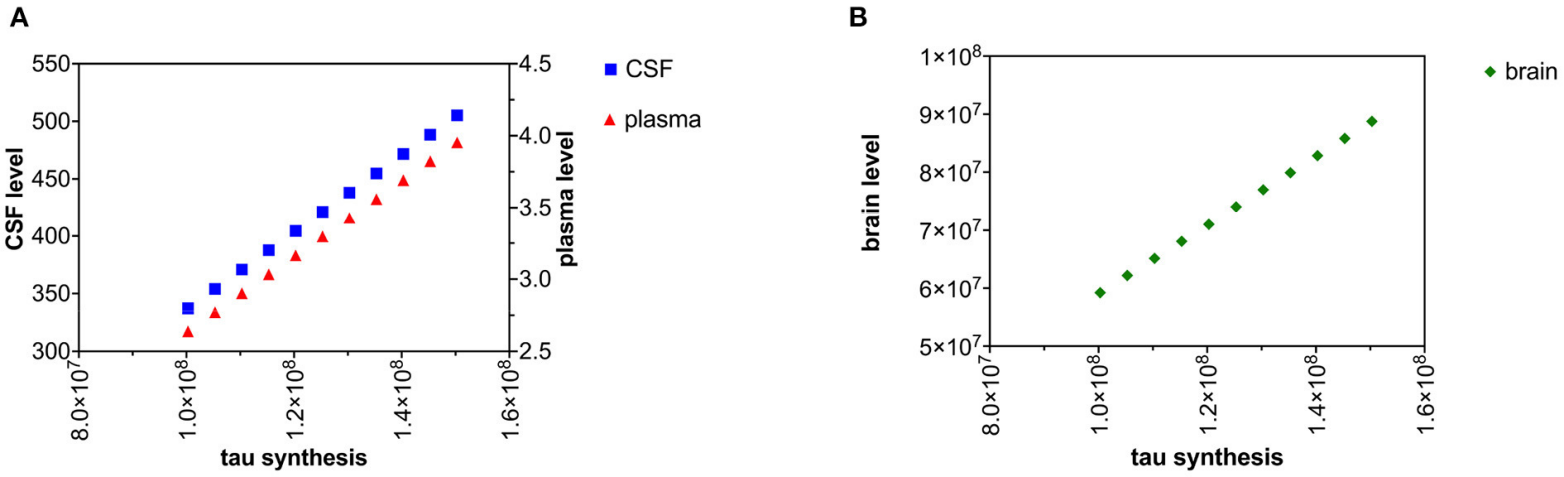
**(A)** Increased neuronal synthesis of tau causes a rise in both CSF and plasma levels of tau. **(B)** Increased neuronal synthesis of tau cause a rise in brain levels of tau.

**Figure 3:**
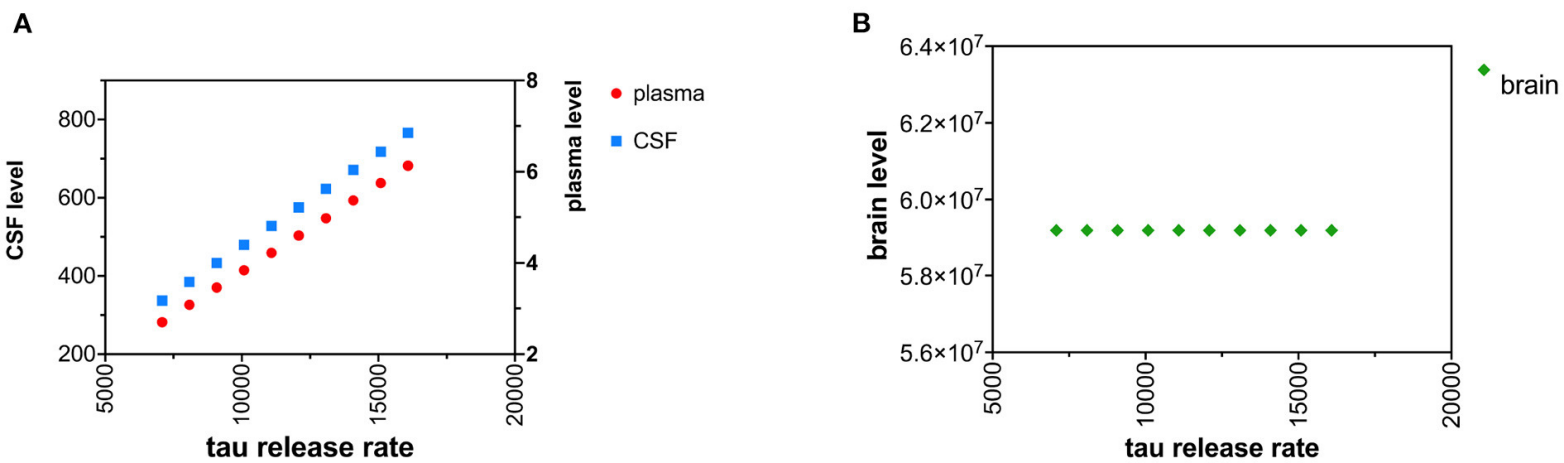
**(A)** Increased neuronal release of tau causes a rise in both CSF and plasma levels of tau. **(B)** Increased neuronal release of tau does not change brain levels of tau.

**Figure 4:**
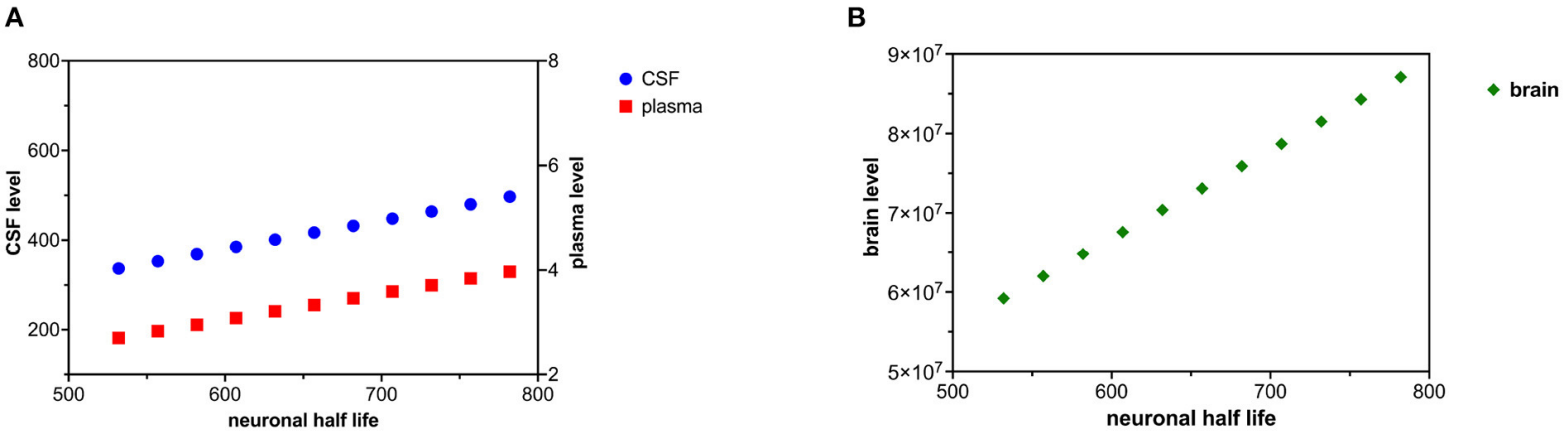
**(A)** Decreased tau neuronal catabolism is reflected as a rise in tau half-life and causes a rise in CSF and plasma levels of tau. **(B)** Decreased neuronal catabolism of tau causes a rise in brain levels of tau.

**Figure 5:**
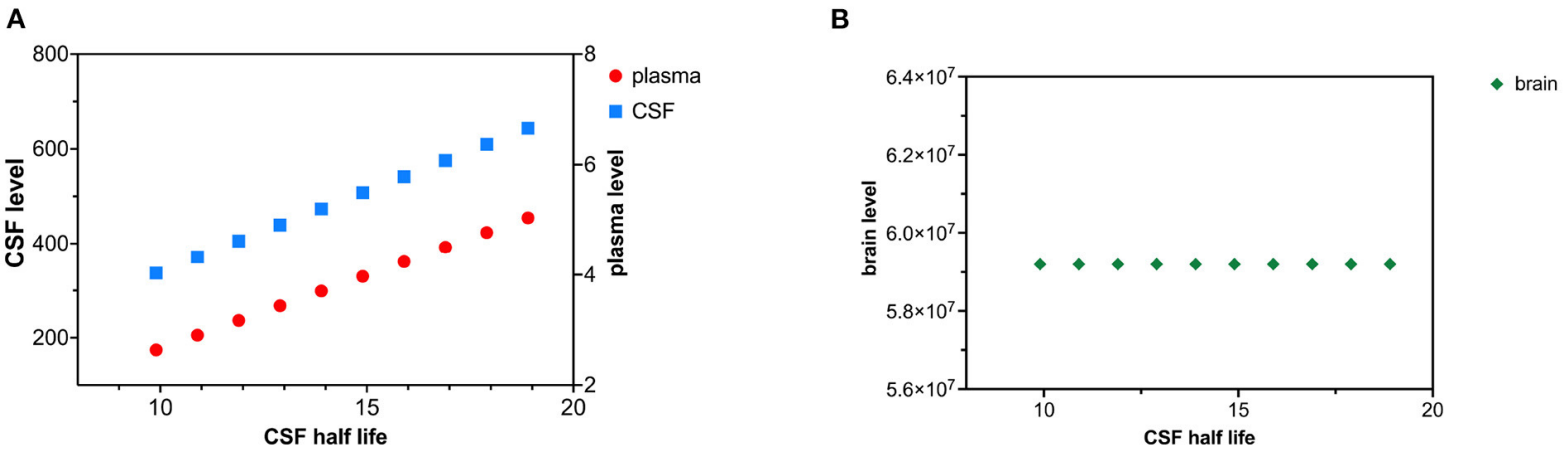
**(A)** Decreased CSF tau clearance as reflected by an increase in tau CSF half-life causes a rise in both CSF and plasma levels of tau. **(B)** Decreased clearance of tau from CSF does not influence brain tau levels.

**Figure 6:**
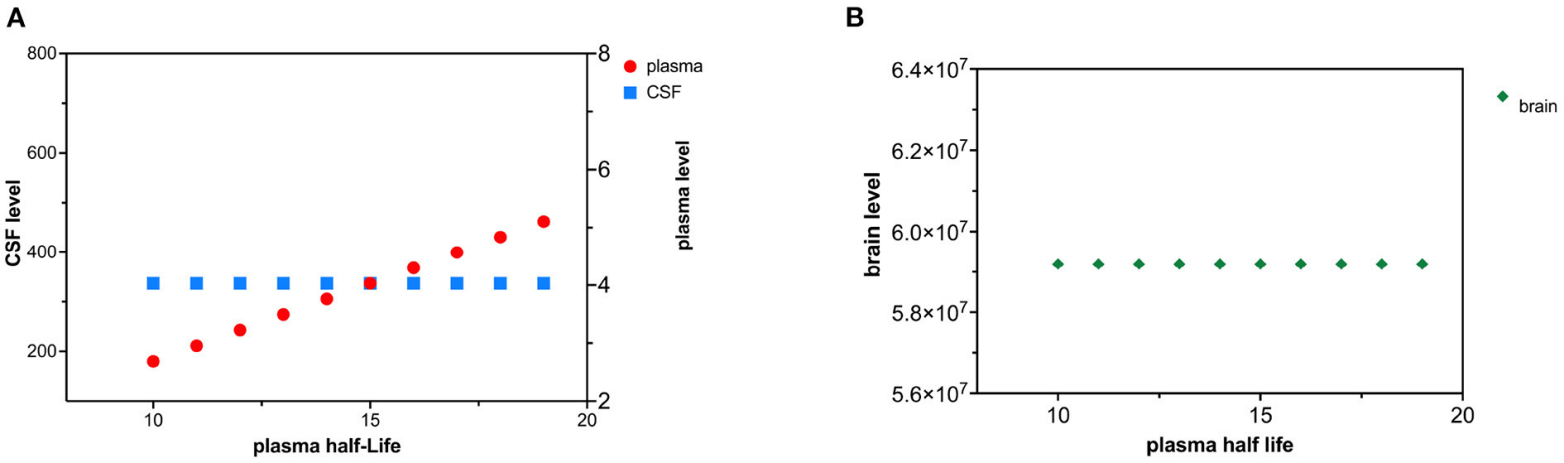
**(A)** Decreased plasma clearance of tau causes a rise in tau plasma levels. **(B)** Decreased plasma clearance of tau does not influence brain tau levels.

* centered Values based on normal values from Table 2. Rates are calculated on a whole brain basis assuming a typical 1,300 g brain with combined 300 ml in CSF-ISF compartment and 3,500 ml in plasma compartment. The kinetic model allows re-calculation of *C*_*brain*_, *C*_*csf*_, and *C*_*plasma*_ in Alzheimer's disease based on alterations in either rates or rate constants (Figures 2A–6B).

† Neuronal half-life based on human isotopic studies (Sato et al., 2018).

‡ By convention, brain size is quantified in g rather than ml.

**Table 2:** Model parameters that vary by disease state.

**Diagnosis**	***C*_*brain*_ ^†^**	***C*_*csf*_ ^‡^**	***C*_*plasma*_ ^†^**	***B*_*brain*_**	***B*_*csf*_**	***B*_*plasma*_**	*F* ^*^
**Units**	**pg/g**	**pg/ml**	**pg/ml**	**pg**	**pg**	**pg**	
Control	5.92*10^7^	337	2.58	7.70*10^10^	1.01*10^5^	9.03*10^3^	0.092
MCI	8.71*10^7^	339	2.71	1.13*10^11^	1.02*10^5^	9.49*10^3^	0.094
AD	8.74*10^7^	403	3.12	1.14*10^11^	1.21*10^5^	1.09*10^4^	0.088

† Level of tau in the brain was expressed as pg of tau per g of brain tissue.

‡ These values should be considered representative as opposed to authoritative.

Brain and CSF levels from Han et al. (2017). Plasma levels based on ADNI cohort (Mattsson et al., 2016). More recent studies show higher values for both CSF and plasma tau (Barthélemy et al., 2020).

* *F* was calculated using (12).

Han et al. (2017) estimated tau as 592.6 ng/mg of brain protein in the temporal lobe of control subjects or 0.059%. The value of *k*_*r*_ was estimated from (14) and values from Tables 1, 2.

## Results

We investigated changes in the brain, plasma, and CSF levels of tau under five scenarios including increased tau neuronal synthesis, impaired tau clearance from the neuron, increased neuronal tau release into the CSF compartment, impaired tau clearance from the CSF compartment, and impaired tau clearance from the plasma compartment. Model results were generated using values from Tables 1, 2 and Equations (7)–(21).

### Tau levels and tau content varies by compartment and disease state

Tau levels are about 120–130 times higher in the CSF than in the plasma (Table 2). Average levels of tau in the brain are 175,000–220,000 times higher than levels of tau in the CSF. Total CSF tau is about ten times higher than total plasma tau. Total brain tau is 10 million times greater than total plasma tau and 1 million times greater than total CSF tau. Tau levels and total tau in the brain, CSF, and plasma of Alzheimer's disease subjects are higher than in control subjects.

### Increased tau neuronal synthesis

Brain tau was estimated as 7.70*10^10^ pg in control subjects (Table 2). A tau half-life in the brain of 552 h (Sato et al., 2018) gives a whole-brain tau synthesis rate of *K*_*synthesize*_ = 9.7*10^7^ pg/hr/brain. We modeled a fixed increase of 5*10^6^ pg/hr/brain in the rate of synthesis of tau over ten time intervals (Figures 2A,B). Brain levels of tau at steady state were calculated by (17) where *K*_*synthesize*_ is the rate of synthesis of tau and *k*_*c*_ is the first-order rate constant for catabolism of tau in the brain compartment. *C*_*csf*_ and *C*_*plasma*_ were calculated with (18) and (19).


(17) $$\begin{array}{l} C_{brain}=\frac{K_{synthesize}}{V_{brain}*k_{c}}. \end{array}$$

(18) $$\begin{array}{l} C_{plasma}=\frac{C_{brain}*V_{brain}*k_{r}*F}{k_{e}*V_{plasma}}. \end{array}$$

(19) $$\begin{array}{l} C_{csf}=\frac{C_{brain}*V_{brain}*k_{r}}{k_{d}*V_{csf}}. \end{array}$$

### Increased tau neuronal release

An increase in the tau release from neurons was modeled as increased tau release of 1,000 pg/hr/brain over 10 time intervals (Figures 3A,B). Although the increased release of tau might be predicted to decrease neuronal levels of tau, due to the small size of the first-order rate constant for release of tau (*k*_*r*_) compared to the first-order rate constant for catabolism of tau (*k*_*c*_) (Table 1), an increase in *k*_*r*_ has a negligible effect on levels of brain tau. *C*_*plasma*_ was calculated with (18). *C*_*csf*_ was calculated with (19).

### Decreased tau neuronal clearance

Decreased tau clearance in the neuron was modeled by an increase in tau neuronal half-life. An increase in tau neuronal half-life corresponds to decreased catabolism (destruction) of tau in the neuron. The neuronal half-life of tau was increased in increments of 25 h over ten time intervals (Figures 4A,B). *C*_*brain*_ was calculated based on (20). *C*_*plasma*_ and *C*_*csf*_ were calculated with (18) and (19).


(20) $$\begin{array}{l} C_{brain}=\frac{K_{synthesis}*0.693}{\text{neuronal half-life}*V_{brain}}. \end{array}$$

### Decreased tau CSF clearance

Decreased clearance of tau from the CSF compartment was modeled as an increase in tau CSF half-life. Altered CSF clearance of tau is not predicted to change brain tau levels. CSF half-life was increased by increments of 1 h over 10 time intervals (Figures 5A,B). *C*_*csf*_ can be calculated from (19) and (21). *C*_*plasma*_ can be calculated from (12) using the calculated value of *C*_*csf*_.


(21) $$\begin{array}{l} k_{d}=\frac{0.693}{\text{CSF half-life}}. \end{array}$$

### Decreased tau plasma clearance

Decreased tau plasma clearance was modeled as an increase in plasma half-life of tau. Tau plasma half-life was increased in 1 hr increments over 10 time intervals (Figures 6A,B). Since the plasma compartment is downstream from the CSF and brain compartment, tau levels in the brain and CSF are not predicted to change. The effect of decreased plasma tau clearance can be calculated using (15) and (18).

## Discussion

### Tau is successively cleared from the brain, CSF, and plasma compartments with different half-lives

Tau has a different elimination half-life in the brain, the CSF, and the plasma (Table 1). The tau literature uses various terms for compartmental elimination including fractional turnover rate, first-order elimination rate constant, elimination clearance, and elimination half-life. If first-order kinetics are assumed, these terms are algebraically related, where FTR is the fractional turnover rate and *k*_*e*_ is the first-order elimination constant.


$$\begin{array}{l} k_{e}=\text{FTR}=\frac{0.693}{\text{elimination half-life}}=\frac{\text{clearance}}{\text{volume of distribution}}. \end{array}$$

Neuronal half-life is the time it takes for neurons to synthesize and replace 50% of their pool of tau. We interpreted the human isotopic labeling study of Sato et al. (2018) as estimating the neuronal half-life of tau as about 23 days. In a mouse model, Fornasiero et al. (2018) found a comparable neuronal half-life of 16 days. In a different mouse model, neuronal tau half-life was ~11 days (Yamada et al., 2011, 2014, 2015). Another mouse model suggests a neuronal half-life of tau of 19.3 days (Kluever et al., 2022).

The plasma half-life of tau reflects the time needed to eliminate 50% of the tau from the plasma (Table 1) and is based on our kinetic model of biomarker levels in mild traumatic brain injury (Azizi et al., 2021). Most elimination of tau from the plasma is likely renal, although elimination by proteolysis and hepatic elimination is possible. Plasma tau levels are elevated in renal failure, consistent with renal elimination of tau (Kitaguchi et al., 2019). We estimated plasma half-life of tau as 10 h but more precise estimates are needed.

The model predicted that at steady state the elimination rate constant for CSF *k*_*d*_ would be equal to the first-order rate constant for absorption of tau into the blood *k*_*a*_ (13). The predicted elimination half-life of tau from the CSF was 9.9 h (Table 1). After minor brain injury, tau and amyloid are rapidly cleared from the CSF with short half-lives (Herukka et al., 2015). Both Lehmann et al. (2019) and Chen et al. (2010) have suggested that protein turnover in the CSF is closely linked to the turnover of the CSF itself. Chen et al. (2010) found a CSF turnover of 10.5 h in sheep. Lehmann et al. (2019) studied the CSF turnover of 197 proteins and estimated a median CSF half-life of 7.4 h based on an isotopic labeling method. When labeled tau is introduced into either the ISF or CSF in mice by injection, it is rapidly cleared from those compartments in < 24 h (Ishida et al., 2022). Our estimate of 9.9 h for tau CSF half-life is at variance with the estimate of 10.9 days of Yamada et al. (2014, 2015) based on a mouse model. In summary, tau is successively cleared from the brain, CSF, and plasma compartments with half-lives of 552, 9.9, and 10 h. These values are subject to revision with more precise measurements.

### Tau is cleared from the neuron primarily by catabolism

An estimated tau neuronal half-life of 552 h gives a whole brain clearance of 1.63 g/h based on a typical 1,300 g brain. With typical brain levels of tau of 5.92*10^7^ pg/g (Han et al., 2017), the tau synthesis rate and the tau clearance rate is at a steady state at 7.44*10^8^ pg/h on a whole brain basis. Tau can be cleared from the cytosol of the neuron by three mechanisms: release to the ISF, internal destruction, or deposition in the neuron as insoluble tau (neurofibrillary tangles). The rate at which tau is converted to neurofibrillary tangles is unknown, but likely to be low compared to the rate of synthesis. Given the low levels of tau in the CSF, we have estimated a first-order tau release rate constant of 9.2*10^−8^ *hr*^−1^ or a tau release rate of 7,080 pg/h on a whole brain basis. Since the tau clearance rate in the neuron exceeds the tau release rate in the neuron by a factor of 10^4^ (Table 1), most tau is cleared by destruction (catabolism) rather than release. Catabolism of tau has been linked to the lysosomal and the proteasomal degradation systems (de Vrij et al., 2004; Chesser et al., 2013; Tarasoff-Conway et al., 2015; Vaz-Silva et al., 2018; Xin et al., 2018; Liu et al., 2021).

In a similar calculation, Han et al. (2017) examined the ratio between soluble tau in the extracellular fluid (CSF) and soluble cytosolic tau. Although they did not do a kinetic analysis, they found that cytosolic brain tau was 345,000 times higher than CSF tau. Han et al. (2017) “hypothesized that the tau secretory process from brain parenchyma to CSF is a passive drainage. This indicates that the secretion factor is most likely proportional to the whole brain parenchymal tau and fits in a typical metabolic first-order kinetic model”. Our model and the data of Han et al. (2017) are consistent with the hypothesis that most cytosolic tau is catabolized and not released into the CSF. This is not surprising given the high energy costs of bringing protein precursors into neurons. It makes energetic sense to recycle misfolded proteins rather than release them into the CSF compartment where they are lost to the blood. Fornasiero et al. (2018) estimated that 50% of new brain protein synthesis involving the essential amino acid lysine utilizes lysine from catabolized brain protein as distinguished from dietary amino acids. This relationship between higher rates of tau catabolism as compared to tau release may not hold under circumstances of neuronal injuries such as traumatic brain injury or stroke.

### Untangling the relative contributions of increased synthesis, decreased catabolism, increased release, and decreased CSF clearance to altered tau levels

Tau levels in the CSF, plasma, and neuronal cytosol are elevated in Alzheimer's disease (Mattsson et al., 2016; Olsson et al., 2016; Han et al., 2017; Koss et al., 2018; Fossati et al., 2019). These higher levels of tau could be due to increased tau synthesis, increased tau release, decreased tau catabolism, decreased CSF clearance of tau, or even decreased plasma clearance.

If we accept that tau levels are increased in all three compartments (brain, plasma, and CSF) in Alzheimer's disease, then decreased plasma clearance is an unlikely explanation for these elevations since decreased plasma clearance of tau is predicted to only increase plasma levels (Figures 6A,B).

Similarly, increased tau release or decreased CSF clearance of tau can explain increased tau levels in the CSF and plasma but cannot explain increased levels of cytosolic tau in the neuron (Figures 3A,B, 5A,B).

Either increased neuronal synthesis of tau (Figures 2A,B) or decreased neuronal catabolism of tau (Figures 4A,B) could account for elevated levels of tau in the brain, CSF, and plasma. Synthesis of tau is likely to be a zero-order kinetic process (independent of cytosolic tau levels) and catabolism of tau is likely to be a first-order kinetic process (linearly related to cytosolic tau levels) (Rosenbaum, 2016). Although there is evidence of increased cytosolic tau in Alzheimer's disease (Han et al., 2017; Koss et al., 2018), evidence of increased synthesis of tau in Alzheimer's disease is lacking. There is growing interest in how tau is catabolized in the Alzheimer's disease (de Vrij et al., 2004; Chesser et al., 2013; Tarasoff-Conway et al., 2015; Vaz-Silva et al., 2018; Xin et al., 2018; Liu et al., 2021). A decline in tau catabolism could explain elevated levels of tau in the brain, CSF, and plasma compartments. A decrease in tau catabolism would be predicted to lengthen tau half-life. In a mouse model, Kleindienst et al. (2010) found 20% longer protein half-lives in aged brains than young brains. An increase in tau synthesis rate would not alter tau half-life, a decrease in tau catabolism would lengthen tau half-life. When Sato et al. (2018) compared tau half-life in 12 Alzheimer subjects to 12 controls, tau half-life was longer in the Alzheimer subjects (31.5 ± 4.0 days) than the control subjects (29.4 ± 6.0 days), consistent with slowed tau catabolism in Alzheimer's disease. However, the difference was not statistically significant.

Based on the findings of this kinetic model and known increases in cytosolic tau, CSF tau, and plasma tau, the most likely underlying mechanisms appear to be decreased neuronal catabolism or increased neuronal synthesis. Animal studies and some human studies are beginning to examine whether decreasing the rate of synthesis of tau in the neuron or enhancing its clearance from the neuron (Bennett et al., 2019; Hoskin et al., 2019; Ossenkoppele et al., 2022) can improve the clinical course of Alzheimer's disease. Kinetic models may prove useful in interpreting animal models that seek to decrease the tau synthesis rate or increase the tau catabolism rate. In animal models, unlike in human studies, it is often possible to measure tau levels simultaneously in the brain, CSF, and blood. For example in an mouse model, when antisense oligonucleotides were used to selectively inhibit tau synthesis in the brain (DeVos et al., 2013), tau levels in the brain and CSF fell in tandem as predicted by our kinetic model.

### Model limitations

The proposed model has several limitations. The model suggest that tau moves from a brain compartment to a CSF compartment to a plasma compartment (Figure 1), a path taken by only minute amounts of cytosolic tau. Precise values for the volumes of distribution in each compartment were not available. We did not correct the plasma volume for sex, weight, or height (Nadler et al., 1962). We combined the ISF and CSF into a single CSF compartment, although tau levels are known to be higher in the ISF than the CSF (Herukka et al., 2015). Our estimate of the volume of distribution of tau in the CSF compartment (*V*_*csf*_) was approximate and was further complicated by tau level gradients within this compartment (Pyykkö et al., 2014).

In addition, we lacked accurate estimates of the tau levels throughout the brain compartment (Han et al., 2017). We have assumed that tau is released, catabolized, absorbed, and eliminated by first-order kinetics and that synthesis follows zero order kinetics (Holford, 2016; Rosenbaum, 2016; Ross et al., 2021). We did not explore physiology-based-pharmacokinetic models or multi-compartment models. The assumption of first order kinetics and linked one-compartment models needs to be supported by additional data.

Our estimates of the first-order plasma elimination constant (*k*_*e*_) and the absorption constants (*k*_*a*_) are subject to error and based on kinetic studies of biomarker levels after mild traumatic brain injury (Azizi et al., 2021). The model could be improved by better estimates of these absorption and elimination constants. We have assumed steady state conditions exist for the blood and CSF tau levels (Rosenbaum, 2016). Although this assumption is reasonable in the short run (Blennow et al., 2007; Slats et al., 2012; Le Bastard et al., 2013), the blood and CSF levels of tau rise with aging (Blomberg et al., 2001; Chiu et al., 2017), and with Alzheimer's disease progression (Palmqvist et al., 2019). There may be diurnal changes in the levels of tau in the CSF (Holth et al., 2019). Another limitations of this study is that we did not account for peripheral (non-brain) sources of tau (Dugger et al., 2016; Barthélemy et al., 2020; Toombs and Zetterberg, 2020).

Barthélemy et al. (2020) have suggested that the lack of correlation between total tau in the CSF and plasma is due to a high contribution of peripheral sources of tau to total tau in the plasma. We did not take into account peripheral sources of tau. Another limitation of this study is that we only looked at total tau (t-tau) and did not consider the kinetics of phosphorylated tau or truncated forms of tau (Barthélemy et al., 2019, 2020).

Our estimate of F, the fractional absorption ratio or bioavailability of tau, is approximate. We estimated a value of 9.2% for *F* (Table 1) meaning only 9.2% of tau in the CSF compartment makes it to the plasma compartment. Although, Nimmo et al. (2020) have suggested that some tau draining from the CSF and ISF gets stuck in macrophages along the drainage pathways, this high loss rate is not fully explained. We did not take into account that some tau may leak from the plasma compartment for redistribution to the body interstitial fluid. Correcting for tau redistribution could generate a higher estimate for F.

The kinetics of the aggregation of tau monomers into oligomers and their eventual deposition into neurofibrillary tangles is of great interest (Kuret et al., 2005; Meraz-Ríos et al., 2010; Meisl et al., 2021), but was outside the capabilities of our model (see also *K*_*nft*_ in Figure 1). Meisl et al. (2021) have described the kinetics governing tau seeds that lead to neurofibrillary tangles in terms of seed growth, seed multiplication, and seed spread. Seed multiplication in Alzheimer's disease appears to be a slow kinetic process with a doubling time as long as 5 years (Meisl et al., 2021). Furthermore, we did not consider alternative models of Alzheimer's disease that model the topographic spread of insoluble tau (neurofibrillary tangles) through the brain (Weickenmeier et al., 2018; Fornari et al., 2019; Cornblath et al., 2021).

Despite these limitations, kinetic equations provide a model of tau content and levels in the brain, CSF, and plasma. Multiple anti-tau clinical trials are in progress for Alzheimer's disease (Ossenkoppele et al., 2022). Most tau is catabolized in the neuron and re-used to make new proteins. A minute amount of tau is released from neurons and successively enters the CSF and plasma compartments. A better characterization of the rates at which tau is synthesized, catabolized, and released from neurons is needed as well as the rates at which tau is cleared from the CSF and plasma. Observed elevations in tau levels in the brain, CSF, and plasma in Alzheimer's disease are most consistent with decreased tau catabolism or increased tau synthesis at the neuronal level.

## Data availability statement

The raw data supporting the conclusions of this article will be made available by the authors, without undue reservation.

## Author contributions

DH and SA: concept and design, data acquisition, model parameters, and computations. All authors contributed to data interpretation, drafting, revising, and approved the submitted version.

## Funding

Research was partially sponsored by the Mary K. Finley Missouri Endowment, the Missouri S&T Intelligent Systems Center, the National Science Foundation and the Leonard Wood Institute in cooperation with the U.S. Army Research Laboratory and was accomplished under Cooperative Agreement Number W911NF-14-2-0034.

## Conflict of interest

The authors declare that the research was conducted in the absence of any commercial or financial relationships that could be construed as a potential conflict of interest.

## Publisher's note

All claims expressed in this article are solely those of the authors and do not necessarily represent those of their affiliated organizations, or those of the publisher, the editors and the reviewers. Any product that may be evaluated in this article, or claim that may be made by its manufacturer, is not guaranteed or endorsed by the publisher.

## Author disclaimer

The views, opinions, findings, recommendations or conclusions contained in this document are those of the authors and should not be interpreted as representing the views or official policies, either expressed or implied, of the Leonard Wood Institute, the Army Research Laboratory, the National Science Foundation or the U.S. Government. The U.S. Government is authorized to reproduce and distribute reprints for Government purposes notwithstanding any copyright notation heron.

## References

[B1] Azizi S. Hier D. B. Allen B. Obafemi-Ayayi T. Olbricht G. Thimgan M.. (2021). A kinetic model for blood biomarker levels after mild traumatic brain injury. Front. Neurol. 12:1121. 10.3389/fneur.2021.668606 34295300 PMC8289906

[B2] Bakker E. N. Bacskai B. J. Arbel-Ornath M. Aldea R. Bedussi B. Morris A. W.. (2016). Lymphatic clearance of the brain: perivascular, paravascular and significance for neurodegenerative diseases. Cell. Mol. Neurobiol. 36, 181–194. 10.1007/s10571-015-0273-8 26993512 PMC4844641

[B3] Banay-Schwartz M. Kenessey A. DeGuzman T. Lajtha A. Palkovits M. (1992). Protein content of various regions of rat brain and adult and aging human brain. Age 15, 51–54. 10.1007/BF02435024 1573400

[B4] Barthélemy N. R. Horie K. Sato C. Bateman R. J. (2020). Blood plasma phosphorylated-tau isoforms track CNS change in Alzheimer's disease. J. Exp. Med. 217:e20200861. 10.1084/jem.20200861 32725127 PMC7596823

[B5] Barthélemy N. R. Mallipeddi N. Moiseyev P. Sato C. Bateman R. J. (2019). Tau phosphorylation rates measured by mass spectrometry differ in the intracellular brain vs. extracellular cerebrospinal fluid compartments and are differentially affected by Alzheimer's disease. Front. Aging Neurosci. 11:121. 10.3389/fnagi.2019.00121 31178717 PMC6537657

[B6] Bennett C. F. Krainer A. R. Cleveland D. W. (2019). Antisense oligonucleotide therapies for neurodegenerative diseases. Annu. Rev. Neurosci. 42:385. 10.1146/annurev-neuro-070918-050501 31283897 PMC7427431

[B7] Blennow K. Zetterberg H. Minthon L. Lannfelt L. Strid S. Annas P.. (2007). Longitudinal stability of CSF biomarkers in Alzheimer's disease. Neurosci. Lett. 419, 18–22. 10.1016/j.neulet.2007.03.064 17482358

[B8] Blomberg M. Jensen M. Basun H. Lannfelt L. Wahlund L.-O. (2001). Cerebrospinal fluid tau levels increase with age in healthy individuals. Dement. Geriatr. Cogn. Disord. 12, 127–132. 10.1159/000051246 11173885

[B9] Brophy G. M. Mondello S. Papa L. Robicsek S. A. Gabrielli A. Tepas J. III. (2011). Biokinetic analysis of ubiquitin c-terminal hydrolase-l1 (UCH-L1) in severe traumatic brain injury patient biofluids. J. Neurotrauma 28, 861–870. 10.1089/neu.2010.1564 21309726 PMC3113451

[B10] Brunello C. A. Merezhko M. Uronen R.-L. Huttunen H. J. (2020). Mechanisms of secretion and spreading of pathological tau protein. Cell. Mol. Life Sci. 77, 1721–1744. 10.1007/s00018-019-03349-1 31667556 PMC7190606

[B11] Chai X. Dage J. L. Citron M. (2012). Constitutive secretion of tau protein by an unconventional mechanism. Neurobiol. Dis. 48, 356–366. 10.1016/j.nbd.2012.05.021 22668776

[B12] Chen C. P. Chen R. L. Preston J. E. (2010). The influence of cerebrospinal fluid turnover on age-related changes in cerebrospinal fluid protein concentrations. Neurosci. Lett. 476, 138–141. 10.1016/j.neulet.2010.04.015 20399250

[B13] Chesser A. Pritchard S. Johnson G. V. (2013). Tau clearance mechanisms and their possible role in the pathogenesis of Alzheimer disease. Front. Neurol. 4:122. 10.3389/fneur.2013.00122 24027553 PMC3759803

[B14] Chiu M.-J. Fan L.-Y. Chen T.-F. Chen Y.-F. Chieh J.-J. Horng H.-E. (2017). Plasma tau levels in cognitively normal middle-aged and older adults. Front. Aging Neurosci. 9:51. 10.3389/fnagi.2017.00051 28321189 PMC5337523

[B15] Cicognola C. Chiasserini D. Eusebi P. Andreasson U. Vanderstichele H. Zetterberg H.. (2016). No diurnal variation of classical and candidate biomarkers of Alzheimer's disease in CSF. Mol. Neurodegener. 11, 1–9. 10.1186/s13024-016-0130-3 27605218 PMC5013624

[B16] Cornblath E. J. Li H. L. Changolkar L. Zhang B. Brown H. J. Gathagan R. J.. (2021). Computational modeling of tau pathology spread reveals patterns of regional vulnerability and the impact of a genetic risk factor. Sci. Adv. 7:eabg6677. 10.1126/sciadv.abg6677 34108219 PMC8189700

[B17] Dadas A. Janigro D. (2018). The role and diagnostic significance of cellular barriers after concussive head trauma. Concussion 3:CNC53. 10.2217/cnc-2017-0019 30202595 PMC6093276

[B18] Dadas A. Washington J. Marchi N. Janigro D. (2016). Improving the clinical management of traumatic brain injury through the pharmacokinetic modeling of peripheral blood biomarkers. Fluids Barriers CNS 13:21. 10.1186/s12987-016-0045-y 27903281 PMC5402680

[B19] de Vrij F. M. Fischer D. F. van Leeuwen F. W. Hol E. (2004). Protein quality control in Alzheimer's disease by the ubiquitin proteasome system. Prog. Neurobiol. 74, 249–270. 10.1016/j.pneurobio.2004.10.001 15582222

[B20] DeVos S. L. Goncharoff D. K. Chen G. Kebodeaux C. S. Yamada K. Stewart F. R.. (2013). Antisense reduction of tau in adult mice protects against seizures. J. Neurosci. 33, 12887–12897. 10.1523/JNEUROSCI.2107-13.2013 23904623 PMC3728694

[B21] Dugger B. N. Whiteside C. M. Maarouf C. L. Walker D. G. Beach T. G. Sue L. I.. (2016). The presence of select tau species in human peripheral tissues and their relation to Alzheimer's disease. J. Alzheimer's Dis. 51, 345–356. 10.3233/JAD-150859 26890756 PMC6074044

[B22] Ercole A. Thelin E. Holst A. Bellander B. Nelson D. (2016). Kinetic modelling of serum S100B after traumatic brain injury. BMC Neurol. 16:3. 10.1186/s12883-016-0614-3 27315805 PMC4912776

[B23] Fleischman D. Berdahl J. (2019). Anatomy and physiology of the cerebrospinal fluid, in Ocular Fluid Dynamics (Cham: Springer), 435–450. 10.1007/978-3-030-25886-3_18

[B24] Fornari S. Schäfer A. Jucker M. Goriely A. Kuhl E. (2019). Prion-like spreading of Alzheimer's disease within the brain's connectome. J. R. Soc. Interface 16:20190356. 10.1098/rsif.2019.0356 31615329 PMC6833337

[B25] Fornasiero E. F. Mandad S. Wildhagen H. Alevra M. Rammner B. Keihani S.. (2018). Precisely measured protein lifetimes in the mouse brain reveal differences across tissues and subcellular fractions. Nat. Commun. 9, 1–17. 10.1038/s41467-018-06519-0 30315172 PMC6185916

[B26] Fossati S. Ramos Cejudo J. Debure L. Pirraglia E. Sone J. Y. Li Y.. (2019). Plasma tau complements CSF tau and p-tau in the diagnosis of Alzheimer's disease. Alzheimer's & *Dement*. 11, 483–492. 10.1016/j.dadm.2019.05.001 31334328 PMC6624242

[B27] Gendreau K. L. Hall G. F. (2013). Tangles, toxicity, and tau secretion in ad-new approaches to a vexing problem. Front. Neurol. 4:160. 10.3389/fneur.2013.00160 24151487 PMC3801151

[B28] Han P. Serrano G. Beach T. G. Caselli R. J. Yin J. Zhuang N.. (2017). A quantitative analysis of brain soluble tau and the tau secretion factor. J. Neuropathol. Exp. Neurol. 76, 44–51. 10.1093/jnen/nlw105 28069930 PMC5901078

[B29] Harrison I. F. Ismail O. Machhada A. Colgan N. Ohene Y. Nahavandi P.. (2020). Impaired glymphatic function and clearance of tau in an Alzheimer's disease model. Brain 143, 2576–2593. 10.1093/brain/awaa179 32705145 PMC7447521

[B30] Herukka S.-K. Rummukainen J. Ihalainen J. von Und Zu Fraunberg M. Koivisto A. M. Nerg O.. (2015). Amyloid-β and tau dynamics in human brain interstitial fluid in patients with suspected normal pressure hydrocephalus. J. Alzheimer's Dis. 46, 261–269. 10.3233/JAD-142862 25720406

[B31] Hier D. B. Obafemi-Ajayi T. Thimgan M. S. Olbricht G. R. Azizi S. Allen B.. (2021). Blood biomarkers for mild traumatic brain injury: a selective review of unresolved issues. Biomark. Res. 9, 1–17. 10.1186/s40364-021-00325-5 34530937 PMC8447604

[B32] Hladky S. B. Barrand M. A. (2014). Mechanisms of fluid movement into, through and out of the brain: evaluation of the evidence. Fluids Barriers CNS 11:26. 10.1186/2045-8118-11-26 25678956 PMC4326185

[B33] Holford N. (2016). Absorption and half-life. Transl. Clin. Pharmacol. 24, 157–160. 10.12793/tcp.2016.24.4.157

[B34] Holth J. K. Fritschi S. K. Wang C. Pedersen N. P. Cirrito J. R. Mahan T. E.. (2019). The sleep-wake cycle regulates brain interstitial fluid tau in mice and CSF tau in humans. Science 363, 880–884. 10.1126/science.aav2546 30679382 PMC6410369

[B35] Hoskin J. L. Sabbagh M. N. Al-Hasan Y. Decourt B. (2019). Tau immunotherapies for Alzheimer's disease. Expert Opin. Invest. Drugs 28, 545–554. 10.1080/13543784.2019.1619694 31094578 PMC7169377

[B36] Huebschmann N. A. Luoto T. M. Karr J. E. Berghem K. Blennow K. Zetterberg H.. (2020). Comparing glial fibrillary acidic protein (GFAP) in serum and plasma following mild traumatic brain injury in older adults. Front. Neurol. 11:1054. 10.3389/fneur.2020.01054 33071938 PMC7530818

[B37] Ishida K. Yamada K. Nishiyama R. Hashimoto T. Nishida I. Abe Y.. (2022). Glymphatic system clears extracellular tau and protects from tau aggregation and neurodegeneration. J. Exp. Med. 219:e20211275. 10.1084/jem.20211275 35212707 PMC8932543

[B38] Josephs K. A. (2017). Current understanding of neurodegenerative diseases associated with the protein tau, in Mayo Clinic Proceedings, Vol. 92 (Rochester, MN: Elsevier), 1291–1303. 10.1016/j.mayocp.2017.04.016 PMC5613938 28778262

[B39] Kitaguchi N. Tatebe H. Sakai K. Kawaguchi K. Matsunaga S. Kitajima T.. (2019). Influx of tau and amyloid-β proteins into the blood during hemodialysis as a therapeutic extracorporeal blood amyloid-β removal system for Alzheimer's disease. J. Alzheimer's Dis. 69, 687–707. 10.3233/JAD-190087 31156161

[B40] Kleindienst A. Schmidt C. Parsch H. Emtmann I. Xu Y. Buchfelder M. (2010). The passage of S100B from brain to blood is not specifically related to the blood-brain barrier integrity. Cardiovasc. Psychiatry Neurol. 2010:801295. 10.1155/2010/801295 20671945 PMC2910463

[B41] Kluever V. Russo B. Mandad S. Kumar N. H. Alevra M. Ori A.. (2022). Protein lifetimes in aged brains reveal a proteostatic adaptation linking physiological aging to neurodegeneration. Sci. Adv. 8:eabn4437. 10.1126/sciadv.abn4437 35594347 PMC9122331

[B42] Koss D. J. Dubini M. Buchanan H. Hull C. Platt B. (2018). Distinctive temporal profiles of detergent-soluble and-insoluble tau and aβ species in human Alzheimer's disease. Brain Res. 1699, 121–134. 10.1016/j.brainres.2018.08.014 30102892

[B43] Kuret J. Congdon E. E. Li G. Yin H. Yu X. Zhong Q. (2005). Evaluating triggers and enhancers of tau fibrillization. Microsc. Res. Techn. 67, 141–155. 10.1002/jemt.20187 16103995

[B44] Le Bastard N. Aerts L. Sleegers K. Martin J.-J. Van Broeckhoven C. De Deyn P. P.. (2013). Longitudinal stability of cerebrospinal fluid biomarker levels: fulfilled requirement for pharmacodynamic markers in Alzheimer's disease. J. Alzheimer's Dis. 33, 807–822. 10.3233/JAD-2012-110029 23034521

[B45] Lehmann S. Hirtz C. Vialaret J. Ory M. Combes G. G. Corre M. L.. (2019). *In vivo* large-scale mapping of protein turnover in human cerebrospinal fluid. Anal. Chem. 91, 15500–15508. 10.1021/acs.analchem.9b03328 31730336

[B46] Lei Y. Han H. Yuan F. Javeed A. Zhao Y. (2017). The brain interstitial system: anatomy, modeling, *in vivo* measurement, and applications. Prog. Neurobiol. 157, 230–246. 10.1016/j.pneurobio.2015.12.007 26837044

[B47] Liu Y. Ding R. Xu Z. Xue Y. Zhang D. Zhang Y.. (2021). Roles and mechanisms of the protein quality control system in Alzheimer's disease. Int. J. Mol. Sci. 23:345. 10.3390/ijms23010345 35008771 PMC8745298

[B48] Lönsmann Poulsen H. (1974). Interstitial fluid concentrations of albumin and immunoglobulin g in normal men. Scand. J. Clin. Lab. Invest. 34, 119–122. 10.1080/00365517409050824 4424039

[B49] Mandelkow E.-M. Mandelkow E. (1998). Tau in Alzheimer's disease. Trends Cell Biol. 8, 425–427. 10.1016/S0962-8924(98)01368-3 9854307

[B50] Mandelkow E.-M. Mandelkow E. (2012). Biochemistry and cell biology of tau protein in neurofibrillary degeneration. Cold Spring Harb. Perspect. Med. 2:a006247. 10.1101/cshperspect.a006247 22762014 PMC3385935

[B51] Mattsson N. Zetterberg H. Janelidze S. Insel P. S. Andreasson U. Stomrud E.. (2016). Plasma tau in Alzheimer disease. Neurology 87, 1827–1835. 10.1212/WNL.0000000000003246 27694257 PMC5089525

[B52] Meisl G. Hidari E. Allinson K. Rittman T. DeVos S. L. Sanchez J. S.. (2021). *In vivo* rate-determining steps of tau seed accumulation in Alzheimer's disease. Sci. Adv. 7:eabh1448. 10.1126/sciadv.abh1448 34714685 PMC8555892

[B53] Meraz-Ríos M. A. Lira-De León K. I. Campos-Peña V. De Anda-Hernández M. A. Mena-López R. (2010). Tau oligomers and aggregation in Alzheimer's disease. J. Neurochem. 112, 1353–1367. 10.1111/j.1471-4159.2009.06511.x 19943854

[B54] Merezhko M. Brunello C. A. Yan X. Vihinen H. Jokitalo E. Uronen R.-L.. (2018). Secretion of tau *via* an unconventional non-vesicular mechanism. Cell Rep. 25, 2027–2035. 10.1016/j.celrep.2018.10.078 30463001

[B55] Molina D. K. DiMaio V. J. (2012). Normal organ weights in men: part ii-the brain, lungs, liver, spleen, and kidneys. Am. J. Forens. Med. Pathol. 33, 368–372. 10.1097/PAF.0b013e31823d29ad 22182984

[B56] Molina D. K. DiMaio V. J. (2015). Normal organ weights in women: part ii-the brain, lungs, liver, spleen, and kidneys. Am. J. Forens. Med. Pathol. 36, 182–187. 10.1097/PAF.0000000000000175 26108038

[B57] Moody L. R. Barrett-Wilt G. A. Sussman M. R. Messing A. (2017). Glial fibrillary acidic protein exhibits altered turnover kinetics in a mouse model of alexander disease. J. Biol. Chem. 292, 5814–5824. 10.1074/jbc.M116.772020 28223355 PMC5392575

[B58] Nadler S. B. Hidalgo J. U. Bloch T. (1962). Prediction of blood volume in normal human adults. Surgery 51, 224–232. 21936146

[B59] Naseri N. N. Wang H. Guo J. Sharma M. Luo W. (2019). The complexity of tau in Alzheimer's disease. Neurosci. Lett. 705, 183–194. 10.1016/j.neulet.2019.04.022 31028844 PMC7060758

[B60] Nimmo J. Johnston D. A. Dodart J. MacGregor-Sharp M. T. Weller R. O. Nicoll J. A.. (2020). Peri-arterial pathways for clearance of α-synuclein and tau from the brain: Implications for the pathogenesis of dementias and for immunotherapy. Alzheimer's Dement. 12:e12070. 10.1002/dad2.12070 32782922 PMC7409108

[B61] O'Connell G. C. Alder M. L. Webel A. R. Moore S. M. (2019). Neuro biomarker levels measured with high-sensitivity digital ELISA differ between serum and plasma. Bioanalysis 11, 2087–2094. 10.4155/bio-2019-0213 31829739 PMC7132632

[B62] Olsson B. Lautner R. Andreasson U. Öhrfelt A. Portelius E. Bjerke M.. (2016). CSF and blood biomarkers for the diagnosis of Alzheimer's disease: a systematic review and meta-analysis. Lancet Neurol. 15, 673–684. 10.1016/S1474-4422(16)00070-3 27068280

[B63] Orr M. E. Sullivan A. C. Frost B. (2017). A brief overview of tauopathy: causes, consequences, and therapeutic strategies. Trends Pharmacol. Sci. 38, 637–648. 10.1016/j.tips.2017.03.011 28455089 PMC5476494

[B64] Ossenkoppele R. van der Kant R. Hansson O. (2022). Tau biomarkers in Alzheimer's disease: towards implementation in clinical practice and trials. Lancet Neurol. 22, 726–734. 10.1016/S1474-4422(22)00168-5 35643092

[B65] Palmqvist S. Insel P. S. Stomrud E. Janelidze S. Zetterberg H. Brix B.. (2019). Cerebrospinal fluid and plasma biomarker trajectories with increasing amyloid deposition in Alzheimer's disease. EMBO Mol. Med. 11:e11170. 10.15252/emmm.201911170 31709776 PMC6895602

[B66] Patel T. K. Habimana-Griffin L. Gao X. Xu B. Achilefu S. Alitalo K.. (2019). Dural lymphatics regulate clearance of extracellular tau from the CNS. Mol. Neurodegener. 14:11. 10.1186/s13024-019-0312-x 30813965 PMC6391770

[B67] Pernègre C. Duquette A. Leclerc N. (2019). Tau secretion: good and bad for neurons. Front. Neurosci. 13:649. 10.3389/fnins.2019.00649 31293374 PMC6606725

[B68] Pooler A. M. Phillips E. C. Lau D. H. Noble W. Hanger D. P. (2013). Physiological release of endogenous tau is stimulated by neuronal activity. EMBO Rep. 14, 389–394. 10.1038/embor.2013.15 23412472 PMC3615658

[B69] Price G. Patel D. A. (2020). Drug Bioavailability. StatPearls [Internet]. Available online at: https://www.ncbi.nlm.nih.gov/books/NBK557852/

[B70] Pyykkö O. T. Lumela M. Rummukainen J. Nerg O. Seppälä T. T. Herukka S.-K.. (2014). Cerebrospinal fluid biomarker and brain biopsy findings in idiopathic normal pressure hydrocephalus. PLoS ONE 9:e91974. 10.1371/journal.pone.0091974 24638077 PMC3956805

[B71] Reiber H. (2001). Dynamics of brain-derived proteins in cerebrospinal fluid. Clin. Chim. Acta 310, 173–186. 10.1016/S0009-8981(01)00573-3 11498083

[B72] Rissin D. M. Kan C. W. Campbell T. G. Howes S. C. Fournier D. R. Song L.. (2010). Single-molecule enzyme-linked immunosorbent assay detects serum proteins at subfemtomolar concentrations. Nat. Biotechnol. 28, 595–599. 10.1038/nbt.1641 20495550 PMC2919230

[B73] Rosenbaum S. E. (2016). Basic Pharmacokinetics and Pharmacodynamics: An Integrated Textbook and Computer Simulations. Hoboken, NJ: John Wiley & Sons.

[B74] Ross A. B. Langer J. D. Jovanovic M. (2021). Proteome turnover in the spotlight: approaches, applications, and perspectives. Mol. Cell. Proteomics 20:100016. 10.1074/mcp.R120.002190 33556866 PMC7950106

[B75] Ruan Z. Ikezu T. (2019). Tau secretion. Tau Biol. 1184, 123–134. 10.1007/978-981-32-9358-8_11 32096034

[B76] Rutili G. Arfors K.-E. (1977). Protein concentration in interstitial and lymphatic fluids from the subcutaneous tissue. Acta Physiol. Scand. 99, 1–8. 10.1111/j.1748-1716.1977.tb10345.x 65903

[B77] Sato C. Barthélemy N. R. Mawuenyega K. G. Patterson B. W. Gordon B. A. Jockel-Balsarotti J.. (2018). Tau kinetics in neurons and the human central nervous system. Neuron 97, 1284–1298. 10.1016/j.neuron.2018.02.015 29566794 PMC6137722

[B78] Shetty A. K. Zanirati G. (2020). The interstitial system of the brain in health and disease. Aging Dis. 11:200. 10.14336/AD.2020.0103 32010493 PMC6961771

[B79] Slats D. Claassen J. A. Spies P. E. Borm G. Besse K. T. van Aalst W.. (2012). Hourly variability of cerebrospinal fluid biomarkers in Alzheimer's disease subjects and healthy older volunteers. Neurobiol. Aging 33:831-e1. 10.1016/j.neurobiolaging.2011.07.008 21880396

[B80] Sorci G. Riuzzi F. Arcuri C. Tubaro C. Bianchi R. Giambanco I.. (2013). S100B protein in tissue development, repair and regeneration. World J. Biol. Chem. 4:1. 10.4331/wjbc.v4.i1.1 23580916 PMC3622753

[B81] Strang K. H. Golde T. E. Giasson B. I. (2019). Mapt mutations, tauopathy, and mechanisms of neurodegeneration. Lab. Invest. 99, 912–928. 10.1038/s41374-019-0197-x 30742061 PMC7289372

[B82] Tarasoff-Conway J. M. Carare R. O. Osorio R. S. Glodzik L. Butler T. Fieremans E.. (2015). Clearance systems in the brain-implications for Alzheimer disease. Nat. Rev. Neurol. 11, 457–470. 10.1038/nrneurol.2015.119 26195256 PMC4694579

[B83] Tarazi R. C. Dustan H. P. Frolich E. D. (1969). Relation of plasma to interstitial fluid volume in essential hypertension. Circulation 40, 357–366. 10.1161/01.CIR.40.3.357 5810892

[B84] Thelin E. P. Zeiler F. A. Ercole A. Mondello S. Büki A. Bellander B.-M.. (2017). Serial sampling of serum protein biomarkers for monitoring human traumatic brain injury dynamics: a systematic review. Front. Neurol. 8:300. 10.3389/fneur.2017.00300 28717351 PMC5494601

[B85] Tobias A. Ballard B. D. (2022). Physiology, Water Balance. StatPearls [Internet]. Available online at: https://www.ncbi.nlm.nih.gov/books/NBK541059/31082103

[B86] Toombs J. Zetterberg H. (2020). In the blood: biomarkers for amyloid pathology and neurodegeneration in Alzheimer's disease. Brain Commun. 2:fcaa054. 10.1093/braincomms/fcaa054 32954304 PMC7425323

[B87] Vaz-Silva J. Gomes P. Jin Q. Zhu M. Zhuravleva V. Quintremil S.. (2018). Endolysosomal degradation of tau and its role in glucocorticoid-driven hippocampal malfunction. EMBO J. 37:e99084. 10.15252/embj.201899084 30166454 PMC6187216

[B88] Weickenmeier J. Kuhl E. Goriely A. (2018). Multiphysics of prionlike diseases: progression and atrophy. Phys. Rev. Lett. 121:158101. 10.1103/PhysRevLett.121.158101 30362787

[B89] Welch R. D. Ellis M. Lewis L. M. Ayaz S. I. Mika V. H. Millis S.. (2017). Modeling the kinetics of serum glial fibrillary acidic protein, ubiquitin carboxyl-terminal hydrolase-l1, and S100B concentrations in patients with traumatic brain injury. J. Neurotrauma 34, 1957–1971. 10.1089/neu.2016.4772 28031000 PMC6913786

[B90] Xin S.-H. Tan L. Cao X. Yu J.-T. Tan L. (2018). Clearance of amyloid beta and tau in Alzheimer's disease: from mechanisms to therapy. Neurotoxicity Res. 34, 733–748. 10.1007/s12640-018-9895-1 29626319

[B91] Yamada K. Cirrito J. R. Stewart F. R. Jiang H. Finn M. B. Holmes B. B.. (2011). *In vivo* microdialysis reveals age-dependent decrease of brain interstitial fluid tau levels in p301s human tau transgenic mice. J. Neurosci. 31, 13110–13117. 10.1523/JNEUROSCI.2569-11.2011 21917794 PMC4299126

[B92] Yamada K. Holth J. K. Liao F. Stewart F. R. Mahan T. E. Jiang H.. (2014). Neuronal activity regulates extracellular tau *in vivo*. J. Exp. Med. 211, 387–393. 10.1084/jem.20131685 24534188 PMC3949564

[B93] Yamada K. Patel T. K. Hochgräfe K. Mahan T. E. Jiang H. Stewart F. R.. (2015). Analysis of *in vivo* turnover of tau in a mouse model of tauopathy. Mol. Neurodegener. 10, 1–9. 10.1186/s13024-015-0052-5 26502977 PMC4621881

